# Streptococcic Infection Followed by Purulent Arthritis, Together with Pulmonary Pneumococcic Infection

**Published:** 1918-11

**Authors:** 


***Streptococcic Infection Followed by Purulent Arthritis, together with Pulmonary Pneumococcic Infection.*** By M. F. Rigaux and M. J. Gate. *Bulletin de la Société de Médecine des Hôpitaux de Paris,* Dec. 31, 1917.

The authors had the opportunity of dealing with a complicated case of streptococcic septicemia followed by complete recovery. The clinical picture of the case was as follows.

The disease began by characteristic chill and severe pain in the right side. Temperature 40.7° C. On the next day temperature was still 40° C. Pneumococci were very numerous in the sputum; a pneumonia was diagnosed.

Two days later severe "sore throat" developed, with white spots from which Gram-positive cocci, different from streptococci, were cultured. After this, blood culture, tried on broth, gave pure strains of streptococci; an abscess of fixation was then promoted. No enlargment of the spleen; no albumin; pneumococci always abundant in the sputum. Later on, the left shoulder was inflamed, and painful under pressure. An arthrotomy of the joint was practised. Fibrinous pus, containing polynuclear cells, and from which streptococci were grown, was removed.

The sequelae of the operation proved excellent. The drainage of the joint was efficient, and the general condition of the patient improved most rapidly.

On the whole, this patient suffered from streptococcic infection, occasioning purulent arthritis of the shoulder, combined with a simple " sore throat " and a bilateral pneumonia. The primary infection was probably the streptococcic one. Owing to it, the general resistance of the organism was broken down, thus allowing the subsequent development of pneumococci always existing in the mouth, and a secondary invasion by these micro-organisms.

